# Active Site Determination of Heterogenized Molecular Electrocatalysts

**DOI:** 10.1002/aenm.70759

**Published:** 2026-02-17

**Authors:** Elena Antoniono, Shyam Kishore Kumar, Monique van der Veen, Thomas Burdyny

**Affiliations:** ^1^ Department of Chemical Engineering, Faculty of Applied Sciences Delft University of Technology Delft The Netherlands; ^2^ e‐Refinery Institute Delft University of Technology Delft The Netherlands

**Keywords:** active site determination, cyclic voltammetry, FeTPP, heterogeneous molecular electrocatalysts, ink optimization, porphyrin

## Abstract

As molecular catalysts are increasingly employed in heterogenized systems such as CO_2_ electroreduction, a need arises for more systematic approaches to characterize their preparation, distribution, and activity. Current means of classifying electroactive versus spectator molecules are insufficient, while improvement of ink formulations, depositions, and distributions relies primarily on indirect links to electrochemical performance. In this study, we expand the common utilization of Cyclic Voltammetry (CV) in homogeneous systems toward heterogenized molecular catalyst architectures. We illustrate how, even with porous catalyst layers containing carbon, ionomer, and molecules, a combined redox wave integration and UV‐vis analysis can be used as a tool for designing a reproducible deposition procedure. An in‐depth CV analysis is then used to study the effect of catalyst aggregation and quantify the number of electroactive sites on carbon supports. We show that FeTPP (Iron(III)meso‐tetraphenylporphyrin chloride) gives a non‐linear electroactive response when loading is varied, allowing for the identification of distinct loading regions of insufficient, optimal, and excessive coverage. A FeTPP to Vulcan carbon mass ratio of 0.1 provides the highest number of electroactive species, giving the lowest expected aggregation. Overall, the CV approaches are extendable to any redox‐active catalysts, providing a versatile means of characterizing porous heterogeneous molecular catalyst systems.

## Graphical abstract

Here, we develop a methodology to examine the active site availability of immobilized transition‐metal molecular complexes via cyclic voltammetry. By using varying loadings of the molecular complex FeTPP physically immobilized onto Vulcan Carbon, we show the influences of FeTPP/VC ratios on the total number of active sites. The work further provides general guidelines for ink optimization for redox‐based applications.

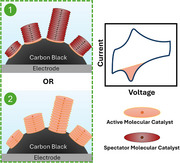

## 1 Introduction

In recent years, CO_2_ electrolysis has been demonstrated as a potentially renewable means of producing of CO and other added‐value products [1, 2, 3]. The most abundantly‐used electrocatalysts are metal bulk catalysts like copper and silver, which contain abundant surface sites capable of activating CO_2_ reduction. A second class of catalysts utilizes highly active sites embedded in a carbon matrix, such as metal‐nitrogen‐carbon single‐atom catalysts. Lastly, transition‐metal molecular complexes, including metalloporphyrins and metallophthalocyanines, provide a more targeted tunability of catalytic behavior due to the flexibility in active site choice and the supporting ligands [4, 5, 6, 7]. Moreover, this class of catalysts has shown potential applications in other areas for sustainable development, such as catalysts for the Oxygen Reduction Reaction, Zinc‐Air Battery Assembly, and CO capturing and separation [8, 9, 10]. Given the broad range of applications of immobilized transition‐metal molecular complexes, there is a need to investigate methods suitable for a range of applications so as to optimize their use.

While metal complexes have been used extensively as homogeneously‐dispersed electrocatalysts in non‐aqueous H‐cell systems, more recent advancements illustrate their viability as heterogeneously‐deposited catalysts capable of CO_2_ conversion in aqueous environments [11, 12]. Importantly, heterogenization allows for the use of molecular catalysts (MC) in gas‐diffusion layer systems such as flow cells and zero‐gap electrolysers [12, 13, 14, 15], which enable higher reactivity due to the proximity to reactive gas phases and removal of gaseous products. Here, metal complexes are typically immobilized via chemical or physical methods onto a conductive surface, such as single or multi‐walled carbon nanotubes, carbon sheets, or nanoparticles [16]. Translating a homogeneous catalyst to heterogeneous systems is not straightforward, however, with a number of added complexities in optimizing catalysts and their activity [11, 17]. For example, heterogeneous systems are typically subjected to aqueous reaction environments and higher current densities, unlike their homogeneous counterparts, which can result in different reaction environments, reaction mechanisms, and surface aggregation of the water‐insoluble catalysts [17].

From a characterization and optimization perspective, the agglomeration, stacking, and crystallization of excess molecular catalysts on an electrode's surface are particularly problematic. Excess material has the potential to not only block active sites but also obscure the location and amounts of electroactive material [18]. Obtaining decisive information on deposited catalysts from common physical characterization techniques such as scanning electron microscopy, X‐ray absorption near‐edge structure (XANES), and X‐ray photoelectron spectrometry (XPS) is limited. In many cases, the optimal ink formulations, catalyst loadings, and deposition procedures for immobilizing catalysts are then indirectly optimized through trial and error using chronoamperometry or chronopotentiometry to determine the most performing system. Inductively Coupled Plasma Optical Emission Spectrometry (ICP‐OES) can also be used to quantify the amount of deposited material, but gives no insight into active versus spectator catalysts [19, 20]. Cyclic Voltammetry (CV), on the other hand, relies on electrochemical signals associated with the reduction and oxidation of the metal center of molecular catalysts and is the predominant characterization method used for homogeneous systems. In principle, CV analyses then provide the most promising means of characterization, as only electroactive materials should elicit a response. With few alternative options, researchers have then adopted CVs as a technique to quantify the amount of electroactive material deposited, and to subsequently compare catalyst performance [6, 21].

**FIGURE 1:**
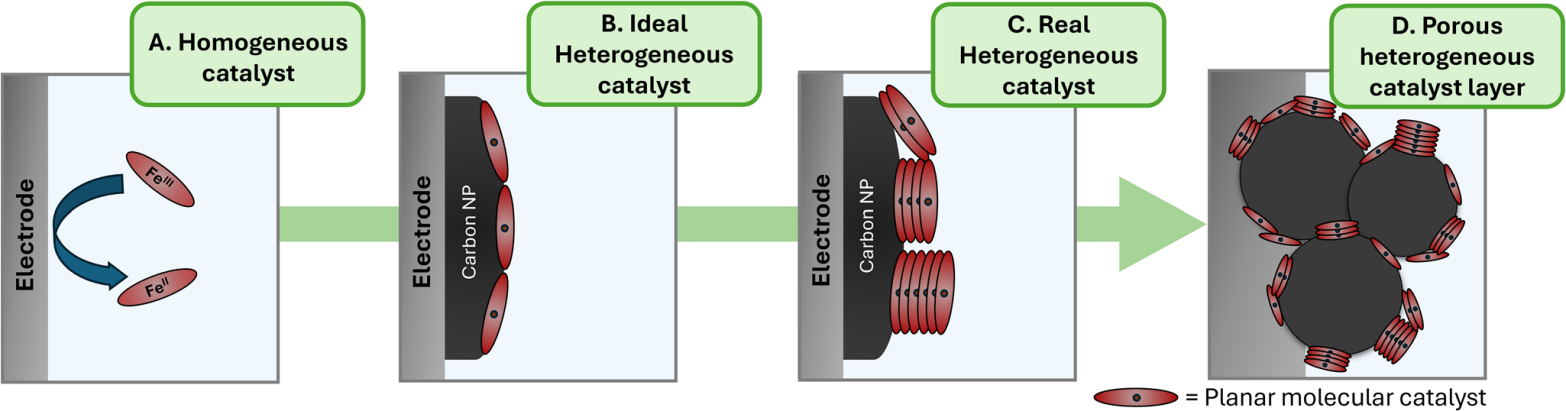
Schematic representation of the decreased quantifiability of Cyclic Voltammetry analysis. Ions and water molecules are omitted from the electrolyte for clarity.

Unfortunately, CV analyses are only ideal and quantitative in planar and thin‐film electrode systems, unlike the 3D porous electrodes typically used for electrolysis [22]. The high internal surface area and subsequent capacitance of the carbon supports can easily obscure molecular catalyst signals, while the lack of semi‐infinite diffusion in the porous layer prevents clear Randles–Ševčík or Nicholson scan rate analyses. Lastly, potential gradients in thicker electrodes lead to broadened redox peaks at higher scan rates due to ohmic drops. The quantitative value of CV analyses then decreases when moving from an ideal homogeneous system to an applied heterogeneous system (Figure 1). With appropriate adaptation and re‐envisioning of cyclic voltammetry, however, we posit CV analyses can remain a powerful and critical tool for the qualitative analysis and reproducibility of heterogeneous systems.

In this study, we probed the use of cyclic voltammetry for characterizing heterogeneous molecular catalyst systems. An unmodified iron tetraphenylporphyrin (FeTPP) complex was chosen as a case study material, given its extensive use in literature and its promising applicability as a catalyst for CO_2_RR [13, 22, 23]. We begin by discussing the use of CV analysis in ink formulation, preparation, and deposition, thus allowing for the reliable production of reproducible catalyst layers of FeTPP immobilized on Vulcan Carbon (VC). Given the wide variety of solvents, binder ratios, and catalyst loadings described in the literature, reproducibility validation analyses are of critical importance. We then show that when varying catalyst loading, a semi‐quantitative CV analysis can be used to determine when catalyst coverage is homogenous on a surface, the degree of aggregation, the electroactive fraction of deposited material, and when the amount of electroactive material is maximized. Our results further conclude that electrochemical activity is restricted to only the first few closest MCs to the carbon support, providing an experimental guideline for how to examine ink optimization and deposition.

## 2 Results and Discussion

### 2.1 Ink Preparation Matters

While the choice of molecular catalyst structure, metal center, and ligands is fundamental to its intrinsic reactivity, the distribution of the catalyst on conductive supports greatly influences its overall activity and stability. The preparation and deposition of the catalyst ink, composed of the molecular catalyst, conductive support, and an ionomer‐containing solvent, is then critical for electrochemical behavior. Numerous successful examples of complex ink formulation procedures using these three components exist in literature [24, 25], but the ratios of components and mixing steps vary widely between sources, as seen in Table 1 [26]. The impacts of varying catalyst to carbon mass ratios, and catalyst to solvent ratios are then unclear, although they are pivotal to the final catalytic architecture.

**TABLE 1:** Key parameters of different ink formulations, taken from literature.

Ink composition	Catalyst: support (mg mg^−1^)	Catalyst: solvent (mg mL^−1^)	Binder: solvent (vol%)	Solvent^a^	Refs.
Modified FeTPP/VC	0.288	1.04	0.002	EtOH	[13]
CoPc/MWCNTs	0.1667, 0.0667, and 0.0333	1	0.029	1:1 EtOH ethylene glycol	[12]
CoPc/CNT	4.3	129	0.01	1:1 EtOH ethylene glycol	[26]
FeF_20_TPP/CNT/CF	0.17	1.7	0.05	7:3 EtOH H_2_O	[27]
FePc/VC	0.0925	0.37	0.01	DMF	[28]
CoPc/MWCNT	1.5	1	—	1:1 DMF IPA	[21]
FeTPP/VC	0.01, 0.1, 0.2, 0.3, 0.4, 0.5, and 1	0.1	0.002	EtOH	This work

^a^ Solvent ratios are given in volume.

Commonly, once an ink has been obtained, the solution is then drop‐casted or spray‐coated on a clean electrode surface, the solvent is evaporated, and the system is successively studied in the preferred electrochemical system. Here, the number of deposition steps and drying procedure are added variables, which are usually not well‐described. To guide the quality and reproducibility of deposited inks we sought to use CV as a primary characterization tool, without needing to explicitly rely on electrochemical performance itself.

**FIGURE 2:**
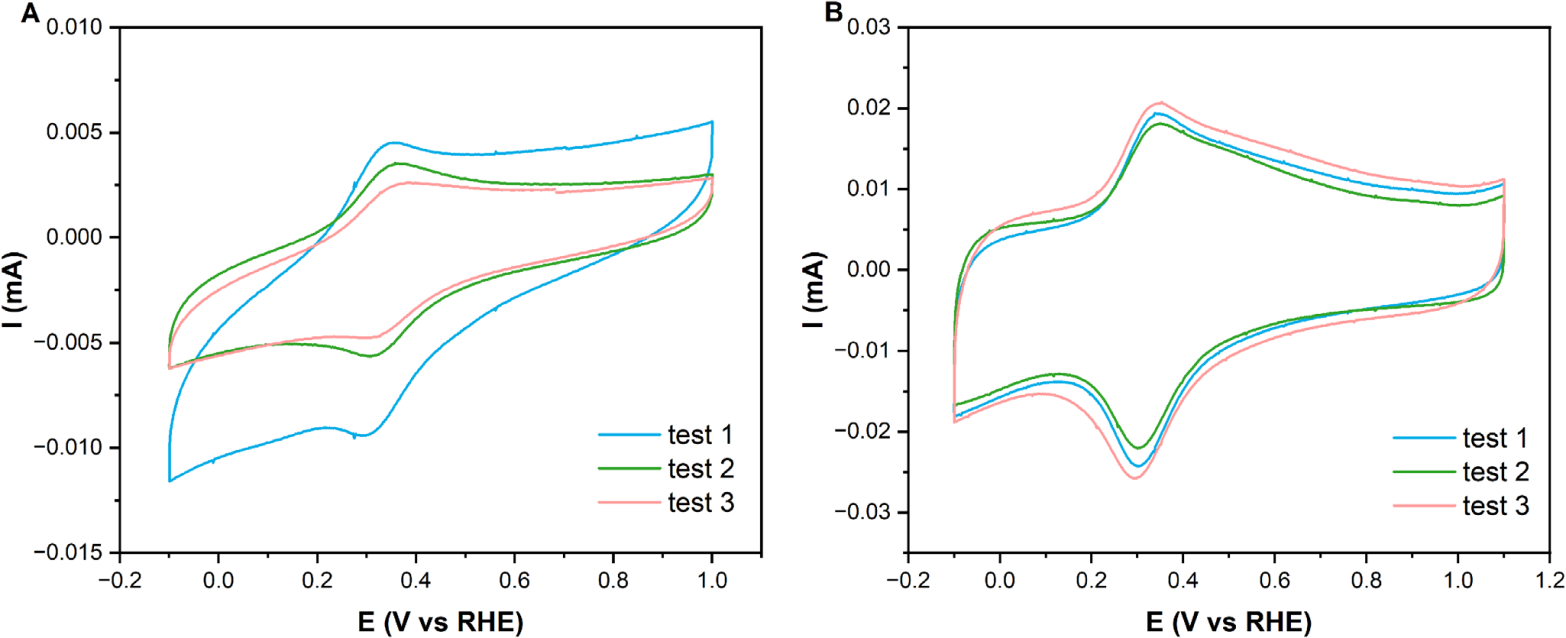
Comparison between the cyclic voltammograms obtained with an unoptimized (A) and an optimized (B) ink preparation procedure. Detailed information on the ink compositions can be found in Tables S1 and S2. The curves were obtained at a scan rate of 1000 mV s^−1^. For each ink, three independent experiments were conducted.

A preliminary CV study targeting reproducibility was performed, using a molecular catalyst ink composed of ethanol solvent, Nafion binder, and a 0.3056:1 mass ratio of FeTPP and Vulcan carbon (see Figures S1–S3 for step‐by‐step ink preparation). A 1 mg mL^−1^ solid to ethanol ratio was then used, for both FeTPP and Vulcan Carbon material, following the ink composition indicated by Torbensen et al., as seen in Table 1 [13]. The ink was then drop cast in a singular step on a polished glassy carbon electrode, allowed to dry, and then analyzed with CV. The process was repeated for three different samples. As shown in Figure 2A, the three samples show considerable divergence in the magnitude and potential of the FeTPP redox waves (between 0.2 and 0.4 V vs. a reversible hydrogen electrode (RHE)). This divergence was also reflected in a visual difference of the samples after drop casting. The different FeTPP suspensions in ethanol prepared as the first step of the ink preparation procedure (Figure S1) also showed slightly different colors, indicating different degrees of molecule solubility. Moreover, the substantial capacitive currents coming from exposed Vulcan carbon make quantitative analysis of the FeTPP redox peaks challenging. These observations led us to conclude that the formation of FeTPP aggregates during the initial dispersion in ethanol was leading to irreproducibility. Additionally, aggregates were hypothesized to form during the drying process, leading to poor coverage of the carbon surfaces even at high loadings, while subsequently limiting the amount of electrochemically active FeTPP.

**FIGURE 3:**
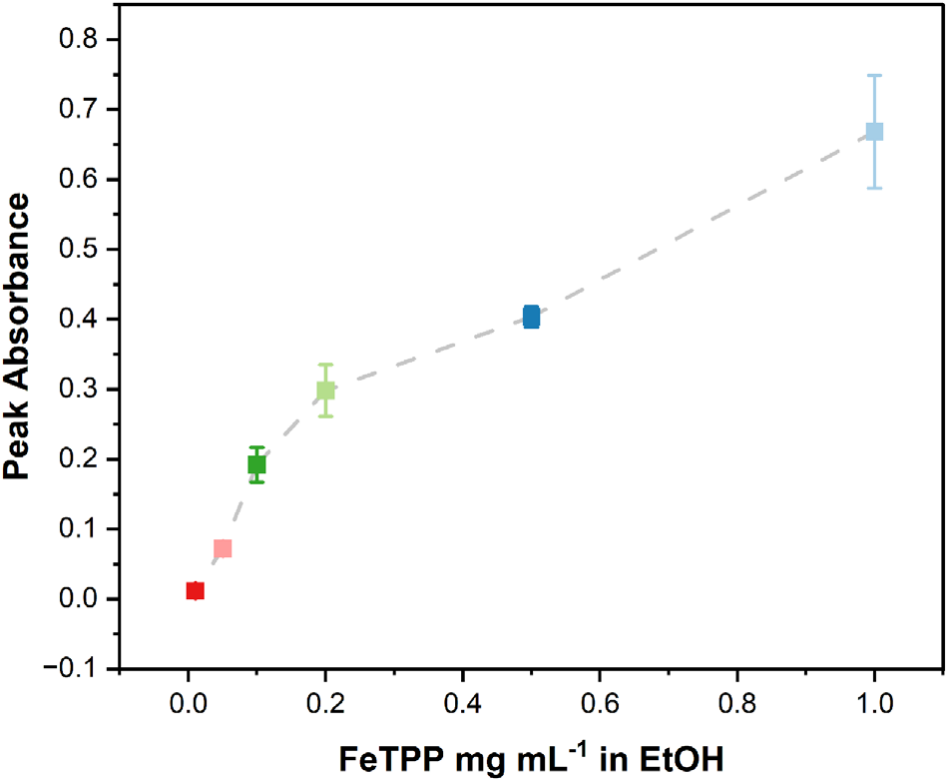
UV‐vis spectroscopy analysis results. Q‐band absorbance at 530 nm, for different FeTPP concentrations in ethanol. Error bars indicate the standard error of the mean of values across three independent experiments. Grey dashed lines are provided as a guide to the eyes, to highlight the observed trends.

An impactful parameter regarding reproducibility was found to be the initial dilution of the molecular catalyst in the chosen solvent (detailed information on the preliminary reproducibility study can be found in Tables S1 and S2). Using UV‐vis spectroscopy, different concentrations from 0.01 mg mL^−1^ to 1 mg mL^−1^ of FeTPP in EtOH were sonicated and studied for monomer quantification in a Lambda 40 Perkin Elmer UV/vis Spectrometer. Here, changes in the Q‐band absorbance are used to quantify the monomer fraction in different FeTPP/EtOH solutions [29]. The FeTPP characteristic Q‐band was identified from literature to be at a wavelength of around 530 nm [30]. As only monomeric catalysts contribute to this absorbance peak, its intensity is expected to increase linearly with the increase in FeTPP concentration, if aggregation is not present [29, 31]. As seen in Figure 3, two different peak intensity trends are readily discernible. Lower concentrations show linearly increasing absorbance, while a sharp decrease in slope is observed at 0.1 mg mL^−1^ FeTPP/EtOH concentration. These results indicate better dispersion and monomerization of the molecular catalyst when using lower FeTPP to ethanol concentration. An initial FeTPP in ethanol concentration of 0.1 mg mL^−1^ was therefore chosen for the optimized ink.

A second critical procedural change was to minimize the drying time of inks, as it can lead to the uneven drying of the solvent, which can result in the formation of the so‐called coffee ring effect [32]. Longer drying times effectively increase ink suspension times, where carbon and FeTPP can agglomerate separately instead of remaining well‐mixed. For ink depositions, it is then critical to use a solvent with a low boiling point, such as ethanol. Even though FeTPP has a greater solubility in dimethylformamide (DMF) than ethanol [28, 33], the higher boiling point leads to extremely long drying times that destabilize the suspension during drying. Lastly, rather than depositing all the prepared ink at one time, the final diluted ink (see Supporting Information for full procedure) was drop‐cast in multiple consecutive steps, with smaller volumes to obtain the final catalyst layer and desired loading. These different factors minimize the drying time, hindering the formation of aggregates. While not shown here, higher temperature and spray‐deposition procedures can also enable shorter drying times, which are more commonly used for planar electrode supports like gas‐diffusion layers.

Following the modification of the ink preparation and deposition procedures, an optimised ink procedure was developed. This procedure resulted in significantly more reproducible CV curves for the final inks deposited on a glassy carbon electrode (Figure 2B). These curves show similar redox wave effects and show reproducible signals at higher scan rates of 1000 mV s^−1^. Shown in Table S2, the redox signal of the optimized ink procedure is also >2‐fold higher than that of the unoptimized case, indicating an increase in electroactive FeTPP.

Another pivotal parameter is the binder concentration, which has various effects, both on the final catalyst electrochemical performance, ink stability, and aggregates formation [25]. In regard to the latter, ionomers serve both as stabilizing and dispersing agents, improving the solid particles adhesion to the electrode surface. It is widely acknowledged that the binder concentration follows a bell‐like trend, with an optimal amount hindering the formation of big aggregates [34, 35]. However, an excessive amount of ionomer in the ink formulation leads to the formation of MC clusters, surrounded by binder long‐chain polymers [34]. For the present study, a concentration of binder equal to the 0.2% in volume of the used solvent was chosen, in line with the ink composition used by Torbensen et al. (Table 1) [13].

Last, an often‐overlooked parameter is the mixing time and mode. For this study, a simple ultrasonic bath mixing procedure was chosen; nonetheless, examples of other techniques, such as ball milling or agar mortar can be found in the literature [26, 36]. A mixing time of 40 min (60 W, 40 kHz) was used for all prepared samples.

Here, we investigated the reproducibility of ink formation and deposition of molecular catalysts using Cyclic Voltammetry. In doing so, we identified drying time and catalyst dispersion concentration as key controlling factors influencing CV reproducibility. Notably, we showed how UV‐vis can be used to avoid aggregation in the initial FeTPP dispersion. With the established approach, we can then extend the CV analysis to more quantitatively assess the electrochemical behavior of the FeTPP catalyst under varying conditions.

### 2.2 Cyclic Voltammetry Signal Interpretation for Active Sites Determination and Maximization

**FIGURE 4:**
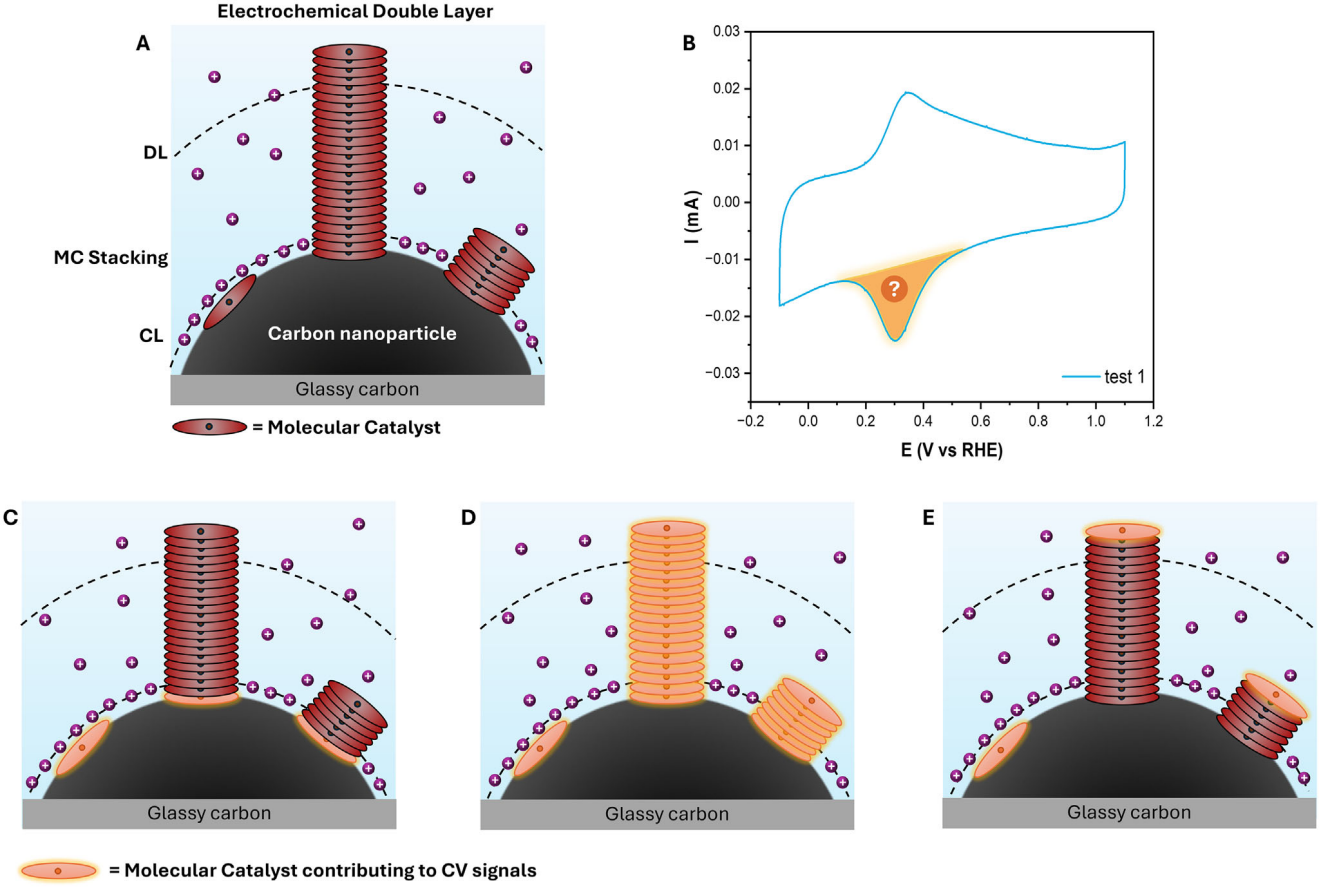
(A) Proposed interfacial structures for the heterogenized FeTPP/VC mixture. Dashed lines indicate the approximate locations of the Diffuse (DL) and Compact layer (CL). Anions and water molecules are omitted from the electrolyte for clarity. (B) Cyclic voltammetry curve obtained for optimized ink composition (in this case FeTPP/VC 0.3, test 1), where the reductive peak response of the metal center Fe(III)/Fe(II) redox couple is highlighted. Schematic representation of different possible cases of activities in a heterogenized FeTPP/VC mixture where redox active molecular catalysts include, (C) only the innermost catalysts closest to the carbon support, (D) multiple molecular catalysts within a π–π stacked layer, or (E) only the outermost catalysts furthest from the carbon support.

In a heterogeneous molecular catalyst system, only a small percentage (on the order of 1%) [13, 21] of the deposited material is typically characterized as electroactive, with the remaining material present as inert aggregates or otherwise inaccessible to electrons from the carbon support. Increasing the electroactive material is not only important for greater activity, but to ensure that excess material does not needlessly hinder the transport of species in the electrolyte. The use of planar molecular catalysts also commonly results in their π‐π stacking on the employed carbon substrate [18, 37, 38, 39, 40], leading to varying thicknesses of non‐active catalyst on the carbon support (Figure 4A). Understanding which metal centers are electrochemically active within these stacked layers then aids catalyst design and optimization. For example, within a stacking of molecular catalysts, will the inner, outer, or multiple metal centers receive and transfer electrons (Figure 4B–E). To try to understand which factors influence electroactive materials and subsequent electrochemical performance, several studies have tested the effect of modifying the MC mass loading, using parameters such as Faradaic Efficiency toward CO production, current density, and stability [33, 34, 35]. However, there are very few studies on the effect of varying molecular catalyst loading on the resulting CV signal [20, 41]. While in the previous section we emphasized reproducibility, here we show the use of redox waves to maximize electroactive species and discern their location relative to the carbon support.

Cyclic Voltammetry of a FeTPP heterogenized molecular system provides information on the oxidation and reduction of the Fe center, as well as an indication of the capacitive currents of the electrode. Observing these redox waves of the metal centers results in duck‐shaped signals, where the peak area is correlated with the amount of charge passed through the metal centers. This amount is then normalized by the scan rate to obtain Q and subsequently divided by the Faraday constant and the number of electrons transferred for the redox event [6, 8, 13, 21]. In principle, for molecular catalysts showing redox‐mediated electrochemical reactions, the measured charge from CV scans can then be used to quantify electroactive catalysts via Equation (1), which can be compared to the known amount of deposited catalyst.

(1) $$\Gamma =Q/\left(\mathrm{nFA}\right)$$

where 𝛤 is the electroactive amount of MC on the modified electrode in mol cm^−2^, *Q* is the integration of the reduction peak (C), n is the number of electrons consumed, *F* is the Faraday constant (96 485 C mol^−1^), and *A* is the geometrical electrode area (cm^−2^) [6, 42, 43]. Detailed information about the procedure followed for the peak analysis and baseline subtraction can be found in Figure S4). Additionally, blank CV analysis of ink containing solely VC and binder solution in ethanol was performed and can be found in Figure S5. This control experiment showed no redox signal in the potential range of interest, indicating that the redox peaks observed in other experiments are to be assigned to the molecular catalyst used.

**FIGURE 5:**
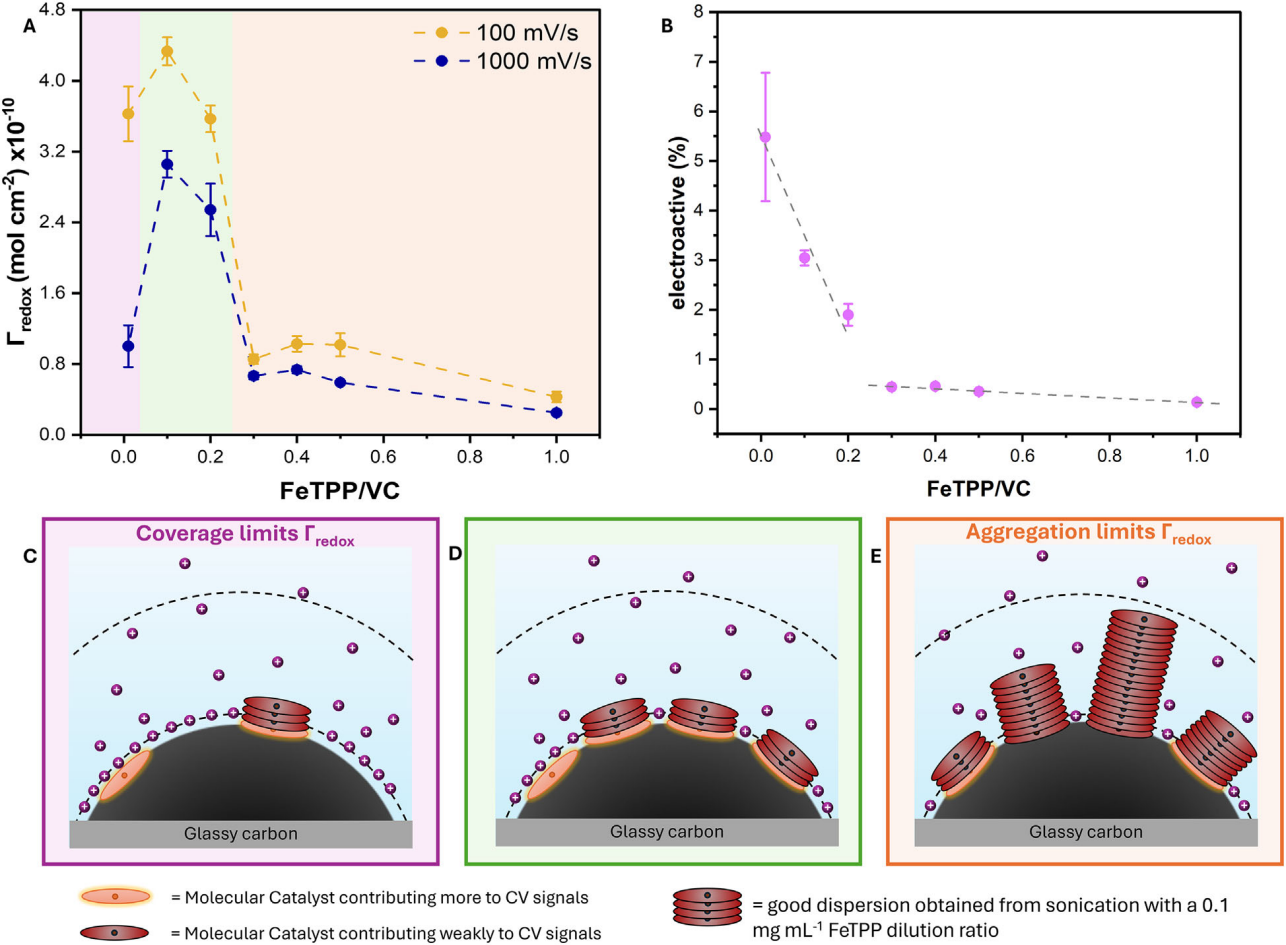
(A) *Γ*_redox_ (mol cm^−2^) across a range of different FeTPP to Vulcan Carbon concentration, for two different scan rates, 1000 (blue curve) and 100 mV s^−1^ (orange curve). All experiments were performed using a 0.5 M NaHCO_3_ electrolyte. Error bars indicate the standard error of the mean of values across three independent experiments. (B) Electroactive fraction of FeTPP for the high scan rate case (1000 mV s^−1^), with different FeTPP/VC amounts. Dashed lines are provided as a guide to the eye, to highlight the observed trends. Schematic representation of the prepared system. FeTPP molecules are shown heterogenized on a Vulcan Carbon surface, which is then immobilized on a Glassy Carbon electrode. (C) low coverage region, from 0.01 to 0.1 FeTPP/VC. D) optimal ink coverage region, from 0.1 to 0.2 FeTPP/VC. E) high loading region, with excess aggregates, representative of ink compositions between 0.3 and 1 FeTPP/VC. Dashed lines indicate the approximate location of the Diffuse and Compact layers. Anions and water molecules are omitted from the electrolyte for clarity.

For this study, different FeTPP to VC substrate ratios were investigated using CV analysis. Cyclic voltammograms were collected using a three‐electrode one‐compartment setup, composed of a glassy carbon working electrode, a platinum coil counter electrode, and a reference Reversible Hydrogen Electrode (RHE). Working electrodes were polished before each experiment with alumina powder on a micro cloth polishing pads, followed by sonication in Milli‐Q water. All experiments were performed using a 0.5 M NaHCO_3_ electrolyte. The electrolyte was bubbled using a nitrogen stream, for two hours before each measurement. Each CV was collected in the potential range between 1 and −0.1 V versus RHE. For each experiment, the measurement was repeated at different scan rates, from 25 to 1000 mV s^−1^, in triplicate. Reproducible signals were obtained for each FeTPP/carbon mass loading studied (Figure S6). A detailed description of the setup used can be found in the Supplementary Information (Figures S3 and S7). The electroactive amount was then calculated by integrating the reductive peak and applying Equation (1). The results obtained are shown in Figure 5A for seven different FeTPP to Vulcan carbon ratios. For each ratio, an adequate amount of sonicated FeTPP in ethanol (0.1 mg mL^−1^) was added to a Vulcan carbon/ethanol solution (1 mg mL^−1^). Aquivion was added to all studied inks, in a fixed 0.2% in volume. A more detailed description of the molecular catalyst ink preparation and deposition can be found in Figures S1 and S2 and Table S3.

As seen in Figure 5A, a nonlinear relationship exists between the decreasing amount of catalyst added and the *Γ*_redox_ calculated. These observations demonstrate the infeasibility of the MC activity behavior described in Figure 4D where all deposited material was considered as undergoing oxidation state change. Three different trends were recognized from the obtained CV analysis data. At high FeTPP loadings, from 0.3 to 1 FeTPP/VC, the amount of electroactive material is relatively constant even with increasing loading. We hypothesize that, within this range of concentrations, FeTPP has covered the whole VC surface, but forms excessive aggregates. The aggregation of FeTPP creates inaccessible stacks that do not give rise to strong CV signals. Notably, the curves obtained for these concentrations result in the lowest variation between triplicates, indicating that excess FeTPP and substantial aggregation itself can lead to reproducible CV curves with proper ink preparation and deposition procedures. Interestingly, lowering the FeTPP/VC ratio from 0.3 to 0.2 results in a drastic increase in the electrochemical signal, from 6.65 × 10^−11^ to 2.54 × 10^−10 ^mol cm^−2^ (Figure 5A). We believe that this sharp variation is due to the concentration of FeTPP being sufficiently low to prevent the formation of aggregates (in crystal structures), and instead to coordinate with carbon and to other FeTPP molecules in π–π stacking. When translating this data to the fraction of electroactive material as shown in Figure 5B, a marked change in both the amount and trend is observed, with electroactive fraction values surpassing 1% for FeTPP mass loadings <0.2 FeTPP/VC. Finally, a different trend was observed when lowering the FeTPP concentration further to 0.01 FeTPP/VC. In this case, a further increase in the electroactive fraction is observed, while the overall redox signal decreases as compared to 0.1 FeTPP/VC. Here the increase in the capacitive signal from the carbon support becomes more prominent (Figure S6A), indicating that the FeTPP loading is insufficient to cover all of the deposited Vulcan carbon. This is highlighted by broader redox peaks and the increased curves capacitance. We then define a loading range between 0.01 and 0.1 FeTPP/VC as necessary to ensure complete carbon coverage. Additionally, it is hypothesized that the total amount of active sites could be improved by increasing the total carbon surface area used, while fixing the optimal catalyst to carbon ratio to minimize aggregation. The proposed methodology could, in fact, be applied to different supports, such as single or multiwalled carbon nanotubes.

Figure 5C–E provide a schematic interpretation of which FeTPP material is electroactive during CV scans, using the data from Figure 5A. We concluded that a 0.1 FeTPP/VC ratio leads to optimized electroactive site (*Γ*_redox_) utilization, equal to 4.34 × 10^−10 ^mol cm^−2^ at 100 mV s^−1^, for the used heterogenized catalytic system. Interestingly, the range of FeTPP/VC leading to higher *Γ*_redox_ was found to be extremely narrow. It is hypothesized that this type of behavior may be exhibited by different molecular catalyst‐based inks, owning to the similarities shared between porphyrin and phthalocyanine complexes, for which π‐π stacking is the predominant immobilization mechanism. Similarly, the described stacking and aggregation mechanisms could be investigated on different carbon substrates commonly used for molecular catalysts physical immobilization, such as carbon nanotubes and Ketjen black [16, 21, 44].

The results described were consistent during analyses performed at different scan rates, as shown in Figure 5A for 100 and 1000 mV s^−1^. However, it is important to remark that *Γ*_redox_ is calculated with Q normalized to the scan rate. Nevertheless, our data show higher electroactive amounts for the analysis at 100 mV s^−1^ compared to higher scan rates of 1000 mV s^−1^. The same behavior was observed in all analyzed inks compositions, regardless of the catalyst's aggregation (Figures S8–S9). The results obtained show how, for all studied ink compositions, lower scan rates give rise to higher electroactive amounts. Interestingly, for all cases, the lowest electroactive amounts are found when applying a high scan rate, with the minimum corresponding to 1000 mV s^−1^ scans. These results highlight how the scan rate selected influences the number of active sites able to undergo oxidation state reduction. This finding provides insight into the mechanism underlying the process of stacked MC activation. As the scan rate is increased, the time scale of the measurement can compete with the time scale of the studied redox reaction [45]. These findings, therefore, highlight a partial kinetic limitation. It is, however, important to underline that, even when applying the lowest scan rate (25 mV s^−1^), the electroactive amounts found do not vary drastically (Figures S8–S9). These results further indicate that only the MC layer in close proximity to the conductive carbon surface is able to undergo an oxidation state change. We therefore suggest CV curves for quantitative analysis to be reported in the literature alongside information on the scan rate used.

Additionally, our results also show that, at most, only the first few layers of deposited molecular catalyst might be electroactive and are responsible for the predominant CV signal response. Otherwise, we would expect loadings of 0.1 and 0.2 FeTPP/VC to be more distinct from each other, rather than exhibiting a similar total amount of electroactive material. The sharp drop in signal at >0.3 loadings suggest that the excess stacked MCs and aggregates are not only spectators electrochemically, but hinder electrochemical activation of the innermost layers. These findings are in contrast with the results of earlier studies [11, 46], which indicate the outermost layer, in close contact with the electrolyte, are primarily responsible for CV signals.

Research dedicated to determining the location of electroactive MCs is ongoing in the literature, which illustrates the complexity of analyzing heterogeneous systems of redox‐active electrocatalysts. In one study Zhu et al. investigated the effect of different‐sized electrolyte cations on an immobilized CoPc system [47], using catalyst monolayers and multilayers. The authors argue that the position of CoPc molecules in the electrochemical double layer (DL) determines the local solvation structure of the adsorbed reaction intermediates, which leads to different product selectivity. Here, the position of the CoPc complexes was given by a different molecular dispersion during the ink preparation procedure, where monolayers exhibited greater methanol production, and stacked structures showed CO_2_ to CO conversion. Here the authors then believe the outermost layer is active and determines selectivity. On the other hand, Jackson et al. showed how, when strong electronic coupling is obtained, the molecular catalyst inner layer, directly in contact with the conductive substrate, is directly responsible for the catalyst CV signal [48, 49]. This scenario is also corroborated by the fact that molecular complexes are considered semiconductors, and the electron transmission mechanism through their metal center and ligands requires substantial overpotentials to drive electrons through stacked MCs [50].

Within this work, we show further support that only innermost MCs are redox active during reductive processes, which can aid in the design and optimization of catalyst layer architectures. We further show a reduction in redox‐active MCs when excess materials are deposited, bringing an added complexity to the deposition processes. Finally, a distinction may exist between deposited materials being redox active and electrochemically active. Specifically, under electrochemical activity, a reactant may also need to be able to diffuse to an active site of an MC, which may further be inhibited by excess materials. Either way, maximizing redox‐active MCs is an essential step to maximizing extrinsic electrochemical activity. Notably, our approach to transition‐metal molecular complexes‐based ink optimization could also be employed in systems not necessarily related to catalytic applications. This is true because CV analysis highlights differences in redox states, which do not by definition correlate to a catalytic event. Consequently, there are many intriguing applications of the present study, outside the realm of reduction reactions, such as the study of MC‐containing inks used as sensor devices and gas capturing [10, 51, 52].

## 3 Conclusion

As molecular catalysts are increasingly employed in heterogenized systems, renewed attention should be paid to the application of traditional electrochemical techniques, such as Cyclic Voltammetry, for their study. When applied thoroughly, CVs can act as a powerful tool for reproducibility, optimization, and scientific understanding. The aim of this study was to assess the reliability of CV‐based analysis for active site determination in heterogenized MC systems. It has been posited that MC aggregation formation leads to an increase in the complexity of the studied system, with a consequent increase in the unreliability of the CV obtained results. Our findings show that it is possible to maximize the electroactive fraction of material through decreased loading, while also identifying the ideal loading threshold for FeTPP to cover the carbon support and maximize the overall amount of electrochemical sites. Other strategies to decrease the molecular catalyst aggregation and increase electrochemically active fractions can be found in the literature, such as covalent grafting and deposition of ultrathin layers [53, 54]. Notably excess immobilized molecular catalyst leads to a plateau of electroactive material and subsequent decrease in the electroactive fraction, demonstrating that only the inner layers of the catalyst are electrochemically active. We conclude that, for a heterogenized FeTPP catalyst on carbon black support, a mass ratio of 0.1 between molecular catalyst and support yields to an optimized electroactive site concentration (4.34 × 10^−10^ mol cm^−2^ at 100 mV s^−1^). This optimum is not to be considered as a universally valid catalyst to support ratio for maximum redox activity, but as an effective example of a novel methodology that can allow this optimum to be found for different systems. This study, therefore sets a framework in which to study different heterogenized systems by employing cyclic voltammetry to optimize their active sites use. Additionally, this study illustrates how CV can be used as the primary technique to investigate heterogenized systems reproducibility and therefore assess the active site quantification results reliability.

## Author Contributions

Conceptualization:Elena Antoniono and Thomas Burdyny. Experimental methodology:Elena Antoniono and Thomas Burdyny. Experimental data and data analysis:Elena Antoniono. Visualization:Elena Antoniono, Shyam Kishore Kumar, Thomas Burdyny. Funding acquisition:Thomas Burdyny. Supervision: Monique van der Veen, Thomas Burdyny. Writing – review & editing:Elena Antoniono, Shyam Kishore Kumar, Monique van der Veen, Thomas Burdyny.

## Conflicts of Interest

The authors declare no conflicts of interest.

## Supporting information




**Supporting File**: aenm70759‐sup‐0001‐SuppMat.pdf

## Data Availability

All data is made available in the manuscript and the supporting information. Raw data and processed data are in tabulated form 4TU.ResearchData.
