# Supplementary material for: Active Site Determination of Heterogenized Molecular Electrocatalysts
Source: Adv Energy Mater. 2026 Feb 17;16(17):e70759. doi: 10.1002/aenm.70759 (PMC13151420; doi:10.1002/aenm.70759)
Supplement: Supplementary file 1 — Supporting File: aenm70759‐sup‐0001‐SuppMat.pdf [file AENM-16-e70759-s001.docx]

**Active Site Determination of Heterogenized Molecular Electrocatalysts**

Elena Antoniono^1,2^, Shyam Kishore Kumar^1^, Monique van der Veen^1,2^, Thomas Burdyny^1,2*^

*Corresponding author email: [T.E.Burdyny@tudelft.nl](mailto:T.E.Burdyny@tudelft.nl)

^1^ Department of Chemical Engineering, Faculty of Applied Sciences, Delft University of Technology, van der Maasweg 9, 2629 HZ Delft, The Netherlands.

^2^ e-Refinery Institute, Delft University of Technology, Leeghwaterstraat 39, 2628 CB Delft, The Netherlands

**Supporting Information**

**Table of Contents**

[1. Optimized ink preparation 1](#_Toc221018748)

[2. Electrode preparation 2](#_Toc221018749)

[3. Electrochemical measurements 2](#_Toc221018750)

[3.1 Reproducibility preliminary study 4](#_Toc221018751)

[3.2 Molecular catalyst loading variation experiments 7](#_Toc221018752)

[References 9](#_Toc221018753)

[**Figure S1.** Schematic representation of the procedure followed for the preparation of the optimized inks used in this article. 1](#_Toc221018754)

[**Figure S2.** A) 0.1 mg mL^-1^ FeTPP in EtOH. No molecular catalyst aggregation was observed, and the solution appeared transparent. B) FeTPP/VC 0.1 ink. 2](#_Toc221018755)

[**Figure S3.** Schematic representation of the one-compartment cell used for all electrochemical experiments. WE: working electrode, modified glassy carbon electrode, CE: counter electrode, platinum wire, RE: reference electrode, Reversible Hydrogen Electrode (RHE). Two needles were used as gas inlet and outlet. 3](#_Toc221018756)

[**Figure S4.** Screenshot taken from *Biologic’s EC-Lab software*, of the CV curve obtained at 1000 mV s^-1^ for test 1 of the ink FeTPP/VC 0.3. Here the potential range chosen for baseline subtraction for all experiments is shown, in grey. 4](#_Toc221018757)

[**Figure S5.** CV curves obtained at 1000 mV s^-1^, for three different amounts of Vulcan Carbon solution (1 mg mL^-1^, in absolute ethanol) drop-casted on the working glassy carbon electrode. All experiments were performed using a 0.5 M NaHCO_3_ electrolyte. 4](#_Toc221018758)

[**Figure S6.** CV curves obtained at 1000 mV s^-1^, for different molecular catalyst to carbon support ratios (FeTPP/VC), in triplicates. For all experiment the optimized ink procedure and composition was used, while changing the catalyst to carbon support ratio. All experiments were performed using a 0.5 M NaHCO_3_ electrolyte. 6](#_Toc221018759)

[**Figure S7**. A) Cyclic voltammetry results of a 0.1 FeTPP/VC sample, at different scan rates. All CV curves were obtained in 0.5M NaHCO_3_ B) Linear relation between the scan rate and the peak current, for a 0.1 FeTPP/VC sample. 7](#_Toc221018760)

[**Figure S8.** Electroactive amount calculated from the integration of the CV curve obtained for different scan rates: 1, 0.5, 0.2, 0.1, 0.05 and 0.025 V s^-1^, for FeTPP/VC equal to 0.1. Error bars indicate the standard error of mean of values across three independent experiments. All experiments were performed using a 0.5 M NaHCO_3_ electrolyte. 8](#_Toc221018761)

[**Figure S9.** Electroactive amount calculated from the integration of the CV curve obtained for different scan rates: 1, 0.5, 0.2, 0.1, 0.05 and 0.025 V s^-1^, for FeTPP/VC equal to 0.1. Error bars indicate the standard error of mean of values across three independent experiments. All experiments were performed using a 0.5 M NaHCO_3_ electrolyte. 9](#_Toc221018762)

## Optimized ink preparation

For the preparation of the optimized ink (used for all different FeTPP/VC loading experiments), 1 mg of FeTPP (Iron (III) meso Tetraphenyl porphyrin chloride, Thermo Fisher Scientific) was added to 10 mL of absolute ethanol (Merck Sigma). This solution (solution A) was sonicated for 40 minutes (HBM machines ultrasonic cleaner, in ‘turbo’ mode). In the same way, 1 mg of Vulcan Carbon (VC, VULCAN XC72 carbon black, CABOT CORPORATION) was added to 1 mL of ethanol and the resulting solution (solution B) was sonicated for 40 minutes. Successively, an appropriate amount of the solution A was added to solution B. This amount was chosen accordingly with the desired final FeTPP to VC ratio, for example, in order to obtain a 0.1 ratio, 1 mL of solution A was added to solution B. Lastly, Aquivion (25±1 % (w/w), 1.14 g mL^-1^, Sigma-Aldrich) was added to the solution, as 0.2% of the total ink volume, which was then sonicated for 30 minutes. The described procedure is summarized in Figure S1. The final obtained ink for the case FeTPP/VC 0.1 is shown in Figure S2B, while Figure S2A shows an example of solution A, obtained for 0.1 mg mL^-1^ FeTPP in ethanol.


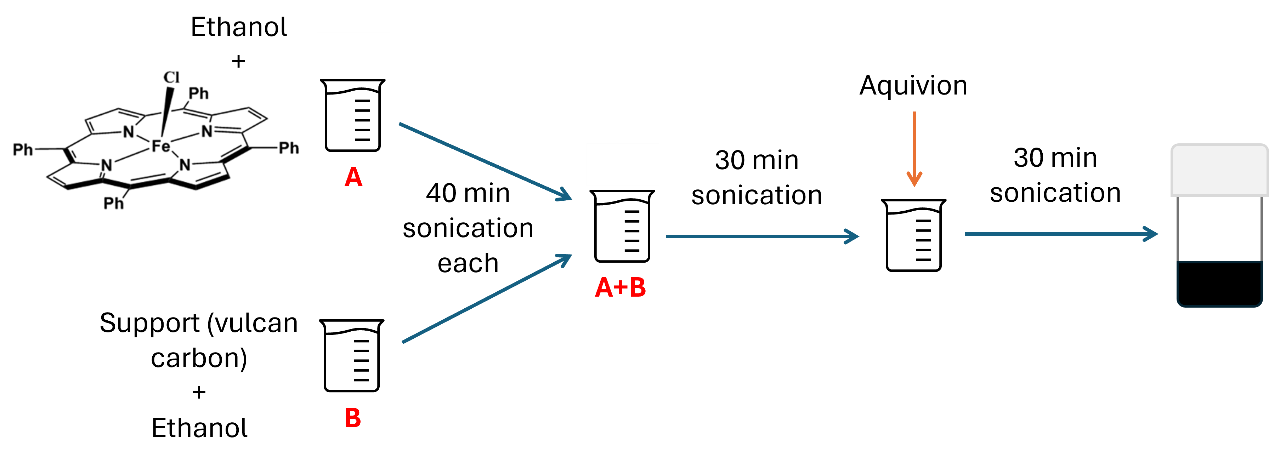


**Figure S1.** Schematic representation of the procedure followed for the preparation of the optimized inks used in this article.


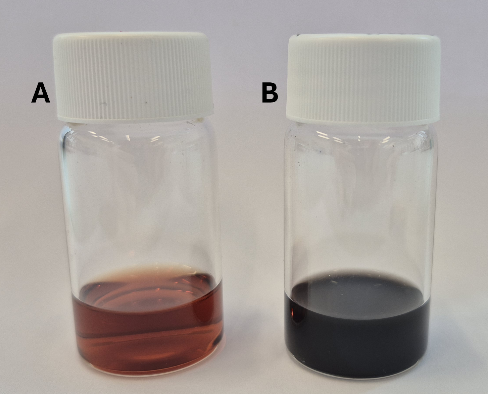


**Figure S2.** A) 0.1 mg mL^-1^ FeTPP in EtOH. No molecular catalyst aggregation was observed, and the solution appeared transparent. B) FeTPP/VC 0.1 ink.

## Electrode preparation

The ink obtained was immediately drop-casted on a clean glassy carbon (Working Disk Electrode - 2 mm diameter, PEEK body 6 mm diameter, Glassy Carbon, 70 mm, Redoxme AB) electrode. All working electrodes were polished before each experiment alternating a cleaning step with alumina powder (0.05 μm and 1 μm, Redoxme AB) on a micro cloth polishing pad and a cleaning step by sonication in a sonicating bath, in Milli-Q^®^ water for 3 minutes. A sequential drop-casting technique was then chosen for all optimized ink depositions. This allowed for a minimization of the drying time and therefore the so-called ‘coffee ring effect’.^1^ For this reason, a total of 8 drops of 5 µL each were deposited on the electrode, for each sample, for a total of 40 µL. The waiting time between each sequential deposition was of 5 minutes. After the final drop-deposition, 30 extra minutes of drying were allowed before starting the electrochemical analysis.

## Electrochemical measurements

All experiments were carried out in a one-compartment cell, consisting of a cylindrical glass container with screw-in cap. Cyclic voltammograms (CV) were collected using a three-electrode setup, composed of a glassy carbon working electrode, platinum coil counter electrode, and a Reversible Hydrogen Electrode (RHE) (Mini-HydroFlex^®^ gaskatel) as a reference. All the electrodes and the needles were inserted in the cell via in-house cut silicone membrane, which allowed for a leak-proof container. The described setup is schematized in Figure S3. All experiments were performed using a 0.5 M NaHCO_3_ electrolyte (Sigma-Aldrich BioXtra, 99.5-100.5%), in which all electrodes were submerged, assuring complete wetting of the catalytic surfaces. The electrolyte was bubbled with a N_2_ stream (Linde, purity 99.999 vol%) for two hours before each measurement. Each CV was collected in the potential range between 1 to -0.1 V vs RHE.^2^ For each experiment the measurement was repeated at different scan rates, from 25 to 1000 mV s^-1^, in triplicates.


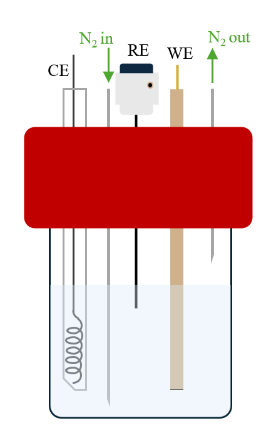


**Figure S3.** Schematic representation of the one-compartment cell used for all electrochemical experiments. WE: working electrode, modified glassy carbon electrode, CE: counter electrode, platinum wire, RE: reference electrode, Reversible Hydrogen Electrode (RHE). Two needles were used as gas inlet and outlet.

All analyses were performed using a SP-200 Potentiostat from Biologic. Each curve was analyzed using Biologic’s EC-Lab software, via their Peak Analysis option, with the Linear Manual mode for the Baseline Correction step. For this correction step, the potential range between 0.12 and 0.48 V vs RHE was selected, for all curves. An example of the result of this correction can be found in Figure S4, for test 1 of the ink FeTPP/VC 0.3.

Figure S5 shows the CV curves obtained for a blank experiment, where different amounts of Vulcan carbon ethanol solution (1 mg mL^-1^, solution B in Figure S1) were sequentially drop-casted on the clean glassy carbon electrode. Also in this case, all inks used contain 0.2% in volume of binder solution, Aquivion, which ensures the ink adhesion to the cathode. All curves obtained show no redox peaks in the potential range of interest.


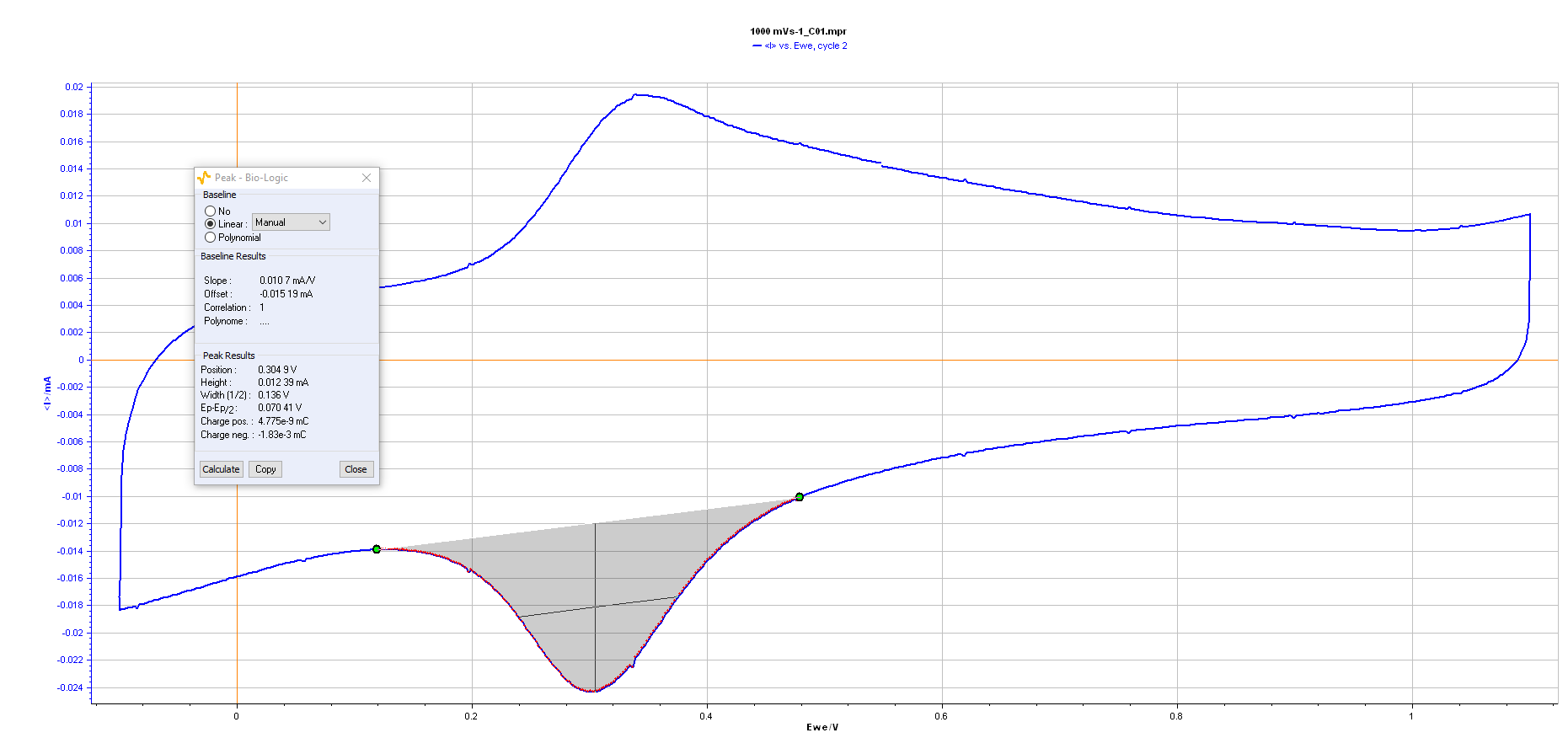


**Figure S4.** Screenshot taken from *Biologic’s EC-Lab software*, of the CV curve obtained at 1000 mV s^-1^ for test 1 of the ink FeTPP/VC 0.3. Here the potential range chosen for baseline subtraction for all experiments is shown, in grey.





**Figure S5.** CV curves obtained at 1000 mV s^-1^, for three different amounts of Vulcan Carbon solution (1 mg mL^-1^, in absolute ethanol) drop-casted on the working glassy carbon electrode. All experiments were performed using a 0.5 M NaHCO_3_ electrolyte.

### Reproducibility preliminary study

Figure 2 in the main text shows the comparison between the cyclic voltammetry curves obtained using unoptimized (Figure 2A) and optimized (Figure 2B) ink procedure. The ink compositions of both unoptimized and optimized case are summarized in Table S1. The main difference in composition is in the FeTPP to EtOH ratio (mg mL^-1^) used in the first step of the ink preparation procedure (solution A in Figure S1). The final optimized FeTPP/EtOH concentration was chosen according to the aggregation study results, conducted using UV-vis spectroscopy analysis (Figure 3). Both inks were prepared and studied as described above. As previously mentioned, in order to decrease the coffee ring effect, for the optimized version a slower drop-casting deposition procedure was adopted. In this case, the drop-casted amount was let to dry completely before continuing with another deposition. On the other hand, for the unoptimized case, the total amount of ink was drop-casted at once. Additionally, in the optimized case Aquivion was selected as binder instead of Nafion (5 wt. %, 0.921 g mL^-1^, Sigma-Aldrich), as a result of the increasing difficulty in purchasing the latter. While the %vol was kept the same in the optimized and unoptimized case, it should be noted that the two binders used have different densities. However, the nature of the two binders is the same, as they both serve as cation conductors. We hypothesize that the different mass percentage used in the two cases would have an impact on the cathodes conductivity, while it should not affect the reproducibility of the measurements. Being the reproducibility of the unoptimized case its major drawback, we deduce the change in binder to not be of pivotal importance for the current study. From the set of different FeTPP/VC ratios analysed in the present study, FeTPP/VC equal to 0.3 was chosen as optimized comparison, given the similarity in molecular catalyst loading on carbon. However, it is important to notice that all optimized FeTPP/VC studies show the same level of reproducibility (see Figure S6).

The parameters characterizing each set of CV curves are summarized in Table S2. The values shown are to be intended as averages of the values obtained for each of the three curves shown. For each parameter, the corresponding Coefficient of Variation (Coeff.V) is shown, which was calculated as a ratio between the standard deviation and the mean. The Capacitive current was calculated as the oxidative current minus the reductive one, at 0.8 V vs RHE. This is to be intended as a measure of the capacitive current obtained, which is strictly related to the carbon material present. The difference in capacitive currents measured in the unoptimized case is an indication of its low reproducibility, linked to both poor ink composition and unreproducible drop-casting. This also reflects in the appearance of the redox peak, which appears diminished. Interestingly, the unoptimized ink also shows unreproducible CV curve skewness, which is an indicator of poor redox reversibility.

**Table S1.** The ink composition of both unoptimized and optimized case.

|  | Unoptimized | Optimized |
| --- | --- | --- |
| FeTPP/VC | 0.3056 | 0.3 |
| FeTPP loading (mg cm^-2^) | 0.0124 | 0.0129 |
| FeTPP/EtOH (mg mL^-1^) | 1 | 0.1 |
| Binder nature | Nafion | Aquivion |
| Binder amount (volume%) | 0.2% | 0.2% |

**Table S2.** Key parameters characterizing each set of CV curve, with their respective Coefficient of Variation (Coeff.V).

|  | Unoptimized | Optimized |
| --- | --- | --- |
| Γ_redox_  (mol cm^-2^) | 9.48×10^−12^ | 6.65×10^−11^ |
| Coeff.V_Γ_redox_ | 23.70% | 8.20% |
| Capacitive current (A) | 3.84×10^−3^ | 1.58×10^−2^ |
| Coeff.V_Capacitive current | 30.02% | 10.29% |
| Peak height (mA) | 1.76×10^−3^ | 1.19×10^−2^ |
| Coeff.V_ Peak height | 24.74% | 5.53% |


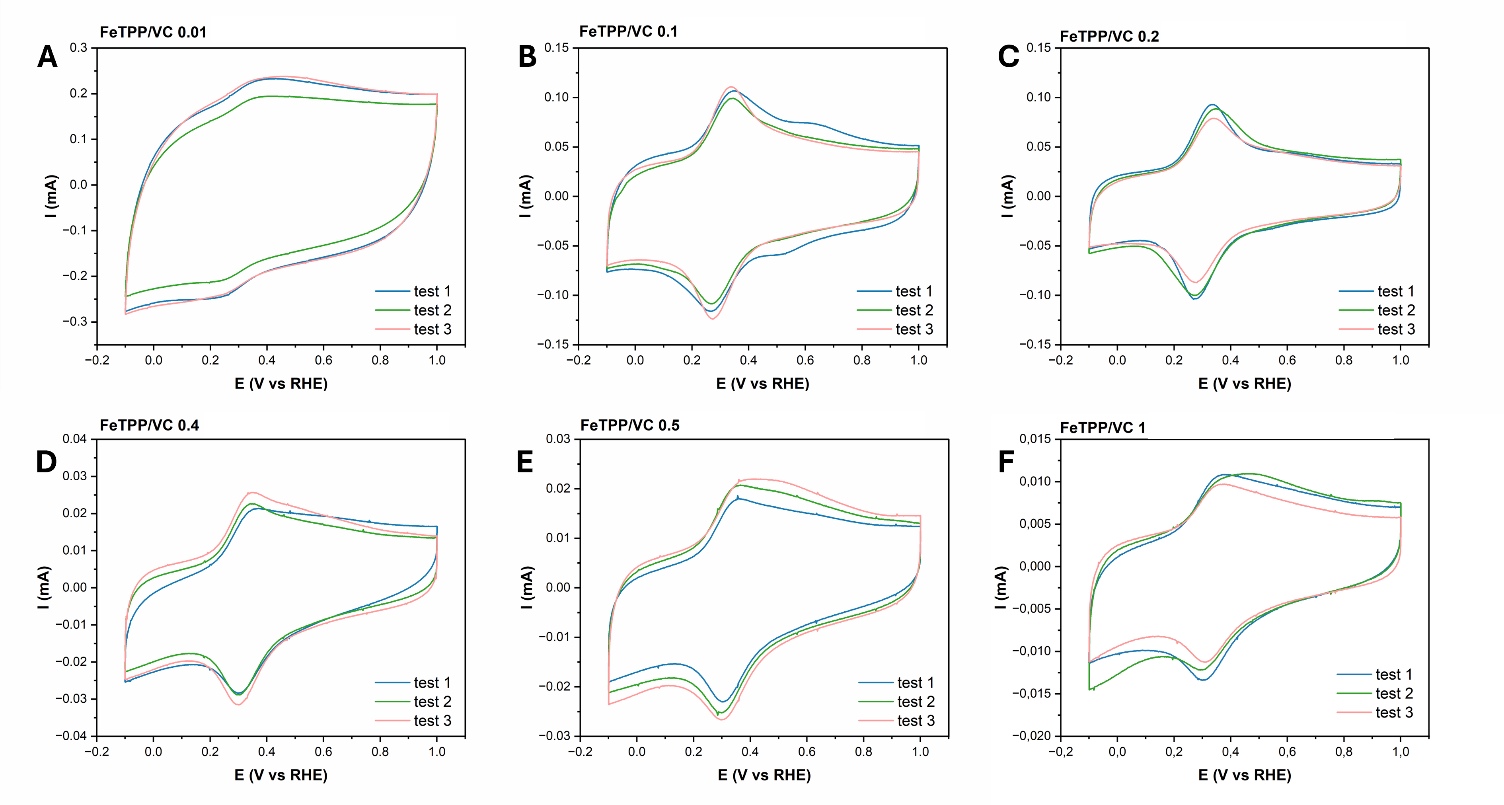


**Figure S6.** CV curves obtained at 1000 mV s^-1^, for different molecular catalyst to carbon support ratios (FeTPP/VC), in triplicates. For all experiment the optimized ink procedure and composition was used, while changing the catalyst to carbon support ratio. All experiments were performed using a 0.5 M NaHCO_3_ electrolyte.

### Molecular catalyst loading variation experiments

All experiments on different molecular catalyst loading were done using the optimized ink composition. Figure S7A shows all curves obtained for a 0.1 FeTPP/VC ink sample. The peak observed at around 0.3 V vs RHE was identified as the Faradaic response of the metal center Fe(III)/Fe(II) redox couple, from literature.^2^ Figure S7B shows the linear relationship between the peak current (I_peak_), for both oxidative (black) and reductive (red) current, and the varying scan rate. The linear variation (dashed lines) indicates the confinement of the redox reaction to the electrode surface.^3,4^


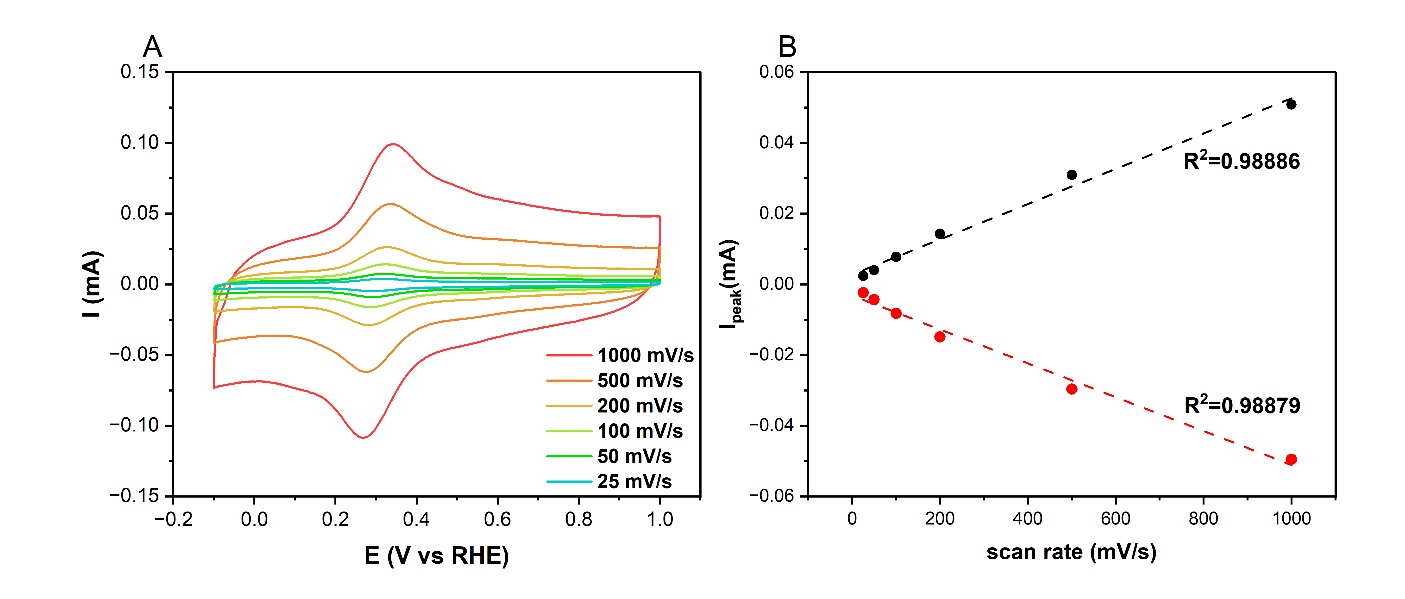


**Figure S7**. A) Cyclic voltammetry results of a 0.1 FeTPP/VC sample, at different scan rates. All CV curves were obtained in 0.5M NaHCO_3_ B) Linear relation between the scan rate and the peak current, for a 0.1 FeTPP/VC sample.

All electroactive fraction of FeTPP for each optimized ink studied, were calculated as:

$Electroactive \left( \% \right)= \frac{\Gamma_{redox}\times A}{{FeTPP}_{deposited}}$ Equation S1

Where $\Gamma_{redox}$ is the electroactive amount calculated from the integration of the obtained cyclic voltammetry peak (mol cm^-2^), A is the electrode area (cm^2^) and ${FeTPP}_{deposited}$ is the amount of catalyst deposited (mol).^5^ Two different linear trends are clearly observed in Figure 5B, with a change occurring between 0.2 and 0.3 FeTPP/VC, signalling a break point where an aggregation limit is identified. Figure S8 and Figure S9 on the other hand show the electroactive amount calculated using Eq. S1 for each FeTPP/VC individually, at different scan rates. Table S3 summarizes all Γ_redox_ calculated from 1000 mV s^-1^ CV analysis, in correlation to the VC and FeTPP deposited amounts, for each of the ink examined.

**Table S3:** Calculated amounts of deposited Vulcan carbon and molecular catalyst for each ink composition used. For each composition, the Γ_redox_ found for 1000 mV s^-1^ is also reported.

| FeTPP/VC | VC deposited (μg) | FeTPP deposited (μg) | Γ_redox_ x10^-10^  (mol cm^-2^) |
| --- | --- | --- | --- |
| 1 | 3.6 | 3.6 | 0.249 |
| 0.5 | 6.7 | 3.35 | 0.591 |
| 0.4 | 8.0 | 3.32 | 0.734 |
| 0.3 | 10 | 3.0 | 0.665 |
| 0.2 | 13.3 | 2.66 | 2.542 |
| 0.1 | 20 | 2.0 | 3.058 |
| 0.01 | 36.3 | 0.36 | 1.001 |


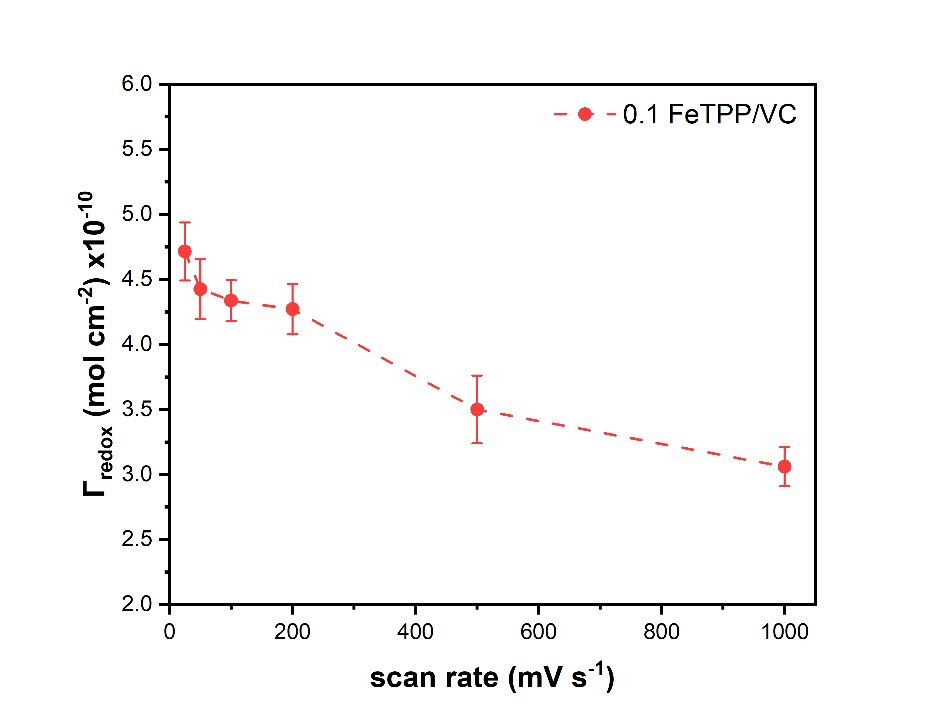


**Figure S8.** Electroactive amount calculated from the integration of the CV curve obtained for different scan rates: 1, 0.5, 0.2, 0.1, 0.05 and 0.025 V s^-1^, for FeTPP/VC equal to 0.1. Error bars indicate the standard error of mean of values across three independent experiments. All experiments were performed using a 0.5 M NaHCO_3_ electrolyte.


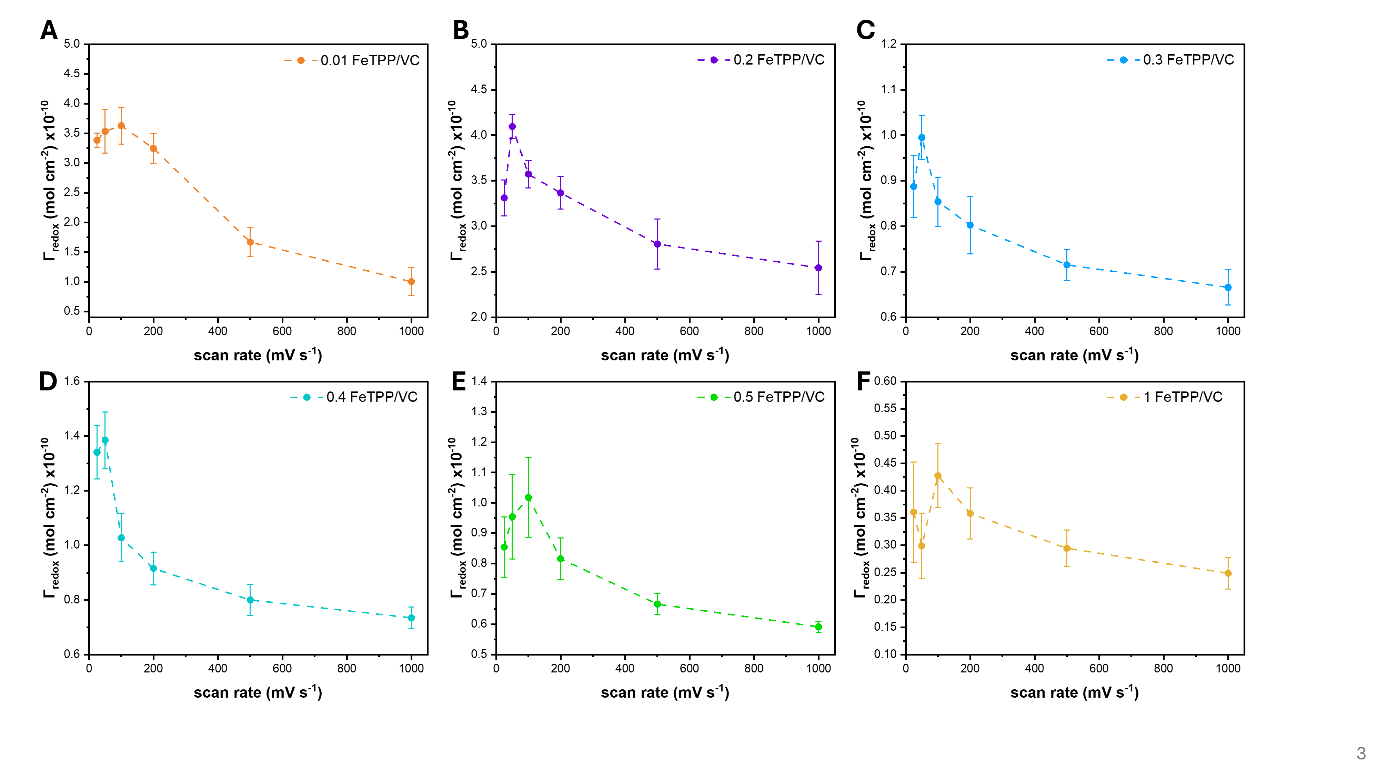


**Figure S9.** Electroactive amount calculated from the integration of the CV curve obtained for different scan rates: 1, 0.5, 0.2, 0.1, 0.05 and 0.025 V s^-1^, for FeTPP/VC equal to 0.1. Error bars indicate the standard error of mean of values across three independent experiments. All experiments were performed using a 0.5 M NaHCO_3_ electrolyte.

## References

1. Kaliyaraj Selva Kumar, A., Zhang, Y., Li, D. & Compton, R. G. A mini-review: How reliable is the drop casting technique? *Electrochem. Commun.* **121**, 106867 (2020).

2. Torbensen, K. *et al.* Iron Porphyrin Allows Fast and Selective Electrocatalytic Conversion of CO _2_ to CO in a Flow Cell. *Chem. – Eur. J.* **26**, 3034–3038 (2020).

3. Allen J. Bard & Larry R. Faulkner. *ELECTROCHEMICAL METHODS Fundamentals and Applications*. (JOHN WILEY & SONS, INC, Department of Chemistry and Biochemistry University of Texas at Austin).

4. Shen, Y., Mu, Y., Wang, D., Liu, C. & Diaconescu, P. L. Tuning Electrode Reactivity through Organometallic Complexes. *ACS Appl. Mater. Interfaces* **15**, 28851–28878 (2023).

5. Chan, T. *et al.* Simple Preparation and Characterization of Hybrid Cobalt Phthalocyanine on Multiwalled Carbon Nanotube Electrodes. *ACS Appl. Energy Mater.* **7**, 2225–2233 (2024).
